# Biological Activity of Sacha Inchi (*Plukenetia volubilis* Linneo) and Potential Uses in Human Health: A Review

**DOI:** 10.17113/ftb.59.03.21.6683

**Published:** 2021-09

**Authors:** Denny M. Cárdenas, Lyz Jenny Gómez Rave, Javier Andrés Soto

**Affiliations:** 1 Universidad de Santander, Facultad de Ciencias Médicas y de la Salud, Grupo de Investigación BIOGEN, Avenida 4 calle 10N-61, 540001 Cúcuta, Colombia; 2 Institución Universitaria Colegio Mayor de Antioquia, Facultad de Ciencias de la Salud, Masira Research Institute, Calle 70 No. 55-210, Bucaramanga, Colombia

**Keywords:** α-linoleic acid, bioremediation, lipid peroxidation, sacha inchi, seed oil tocopherol

## Abstract

Sacha inchi (*Plukenetia volubilis* Linneo) is an ancestral plant originating in the Amazon jungle that has been adopted as a food source due to its high nutritional value, which has gradually been recognized to have potential benefits for human health. Diverse prospective studies have evaluated the effect of consuming components from the plant, derivatives from its seeds, leaves and shell on preventing the risk of cardiovascular disease, chronic inflammatory disease, dermatitis and controlling tumor proliferation, especially given its recognized high content of essential fatty acids, phenolic compounds and vitamin E, showing antioxidant, hypolipidemic, immunomodulation and emollient activity, as well as the capacity to remove heavy metals from aqueous solutions. This review offers a complete description of the existing information on the use and biological activity of *P. volubilis* L., based on its essential lipid components and evidenced on its use in the field of human health, in prevention, therapeutic and nutritional contexts, along with industrial uses, making it a promising bioresource.

## INTRODUCTION

**Fig 1:**
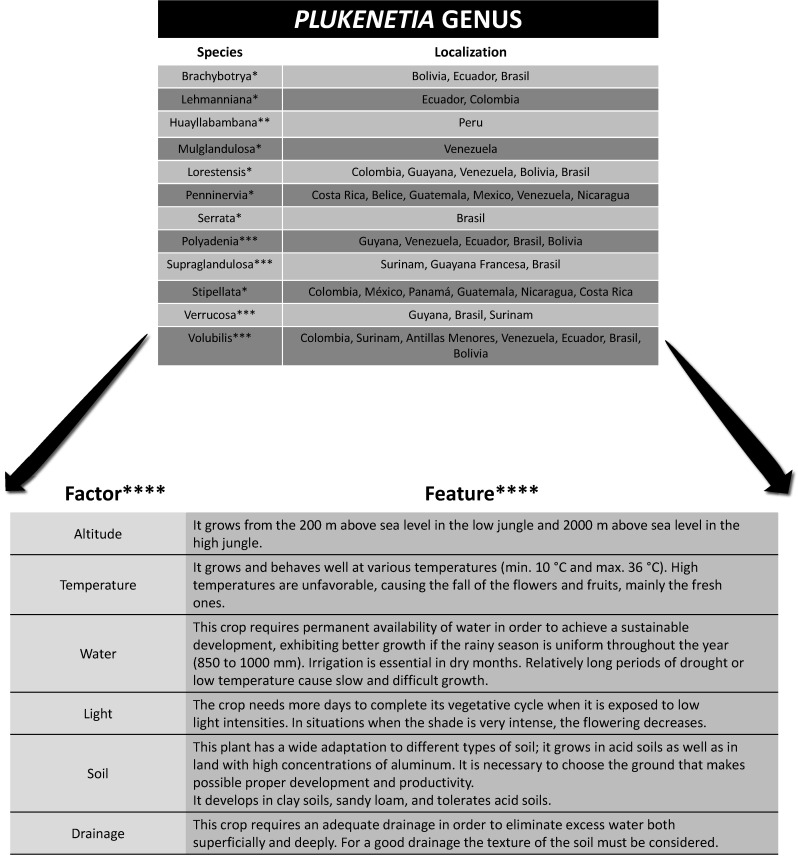
Geographical distribution of the *Plukenetia* genus in Latin America and crop features of the *volubilis* species. Brasil, Venezuela and Colombia are the countries that are host to more genera of the plant due to the extension of the Amazon jungle and therefore to the biodiversity of this region. One of the most striking characteristics of this type of plant is its ability to grow in different types of soils, although it is also true that it requires certain conditions for proper growth, one of them being the constant availability of water sources. Adapted from * (*4*), ** (*5*), *** (*6*) and **** (*7*)

The genus *Plukenetia* has a wide geographical distribution, especially in Central and South America, being present from the Antilles to Bolivia. Sacha inchi (*Plukenetia volubilis* Linneo) is a native plant to the Peruvian jungle that belongs to the Europhorbiaceae family, which encompasses 300 genera and 7500 species (*1*). It is cultivated at an altitude of 200 to 2000 m above sea level (*2*, *3*) and its growth is conditioned by different geoclimatic aspects (Fig. 1) (*4*-*7*).

**Fig. 2:**
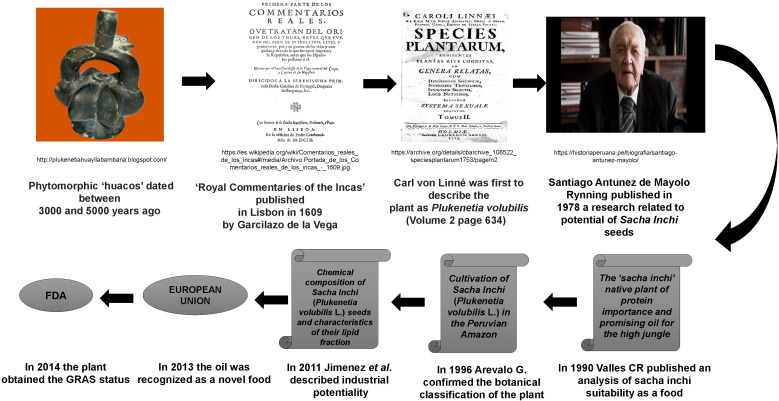
Timeline of historical events of the sacha inchi plant. It is a crop planted by ancient pre-Inca cultures such as the Mochica and Chimú civilizations (*8*), whose main physical and organoleptic characteristics were first described by de la Vega (*9*), and which Linne later called *Plukenetia volubillis* (*10*). In the 1970s, a former Peruvian minister for agriculture screened the potential of the Amazonian region for new types of food crops, thus rediscovering sacha inchi, describing its chemical and nutritional attributes (*10*). Sacha inchi has shown its potential as a source of food between tribes, as a novel food, and the whole plant as drug Generally Regarded As Safe (GRAS) by the US Food and Drug Administration (*11*)

The antiquity of the crops and the importance of this plant are evidenced in the archaeological findings of pre-Inca utensils (*8*), and throughout history several events have brought to the fore the relevance of this plant on a social and industrial level (Fig. 2 (*8*-*11*)). Its common name in the native Quechua language means false (sacha) peanut (inchi), given its use as an edible nut initially by the pre-Inca Chanka and Mochica-Chimú indigenous tribes. It is currently cultivated in Asian countries, like Thailand, China and Vietnam (*3*, *12*-*15*), as well as in Central and South America where, in addition to representing a nutritional alternative, it has become an opportunity for economic development (*12*).

It is known as mountain peanut, sacha peanut or Inca nut (*13*, *14*, *16*). Its seed contains polyunsaturated fatty acids, like α-linolenic (ALA) and linoleic (LA), bases of ω-3 and -6, respectively (*13*), γ- and δ-tocopherols, natural forms of vitamin E, known for its antioxidant activity and antitumor potential (*17*-*22*).

To date, positive outcomes have been recognized from consuming the plant or the components present in the seed, shell and leaves. It is worth highlighting its contribution of energy and proteins, generation of lipid mediators with immunomodulatory activity, regulation of cholesterolaemia, brain function and blood pressure (*16*, *23*-*25*), and antioxidant and antitumor potential (*26*-*30*). Due to the aforementioned, it is relevant to explore in detail the benefits of this Amazonian plant.

The possibility of having access to such benefits is facilitated by the industrial extraction processes, mainly based on treating the oily fraction by using pure liquid CO_2_, obtaining a sample rich in ω-3 without causing a significant impact on the original composition (*31*). Bearing in mind the relevance of the benefits of this promising plant on human health (*32*), this review offers a description of the state of the art regarding sacha inchi, with special emphasis on its biological activity, the characteristics of its components and nutritional usefulness.

## COMPOSITION OF THE PLANT

The sacha inchi seed extract predominantly contains lipids (35-60%), represented by polyunsaturated fatty acids (PUFA) like α-linolenic acid or ALA (C18:3, ω-3; 47-51%) and linoleic acid or LA (C18:2, ω-6; 34-37%) (*25*), in addition to monounsaturated fatty acids like oleic acid (~9.5%), saturated fatty acids like palmitic (4.4%) and stearic (2.7%) acids, proteins (25-33%) and other minor compounds like vitamin E in its α-tocopherol (50-114 mg/g of oil) and δ-tocopherol (30-125 mg/g) forms, flavonoids, secoiridoids, lignans, phenols, campesterol, stigmasterol, β-sitosterol and minerals (*13*, *14*, *17*, *18*, *33*-*36*).

However, sacha inchi PUFA content could be even higher, representing around 93% of the fatty acids, especially at the expense of essential fatty acids, like ALA and LA, a key fact that differentiates it from other oleaginous plants of great interest, like the olive tree, whose oil contains 1% ω-3 and 9% ω-6 fatty acids. This availability seems to vary according to the crop zone, as shown by chromatographic characterization of the oil obtained from certain regions of Ecuador, where linoleic (C18:2, ω-6) and linolenic (C18:3, α-isomer, ω-3) acids constitute the majority of the lipid component, with the latter being even significantly greater than that registered for oil from other plant species, like palm, corn, soy and sunflower (*24*). Likewise, the compositional analysis of the sacha inchi seed cultivated in the department of Putumayo, Colombia, reveals a predominantly lipid content ((42.75±0.5) %), within the range described for the species of Peruvian origin, where 83.3% of these corresponds to polyunsaturated fatty acids, with a very similar content of monounsaturated (9.4%) and saturated (7.3%) fatty acids, as well as protein ((29.85±0.085) %) (*23*).

The content of unsaturated fatty acids in the lipid component of the seeds also varies inversely with temperature; thus, the analysis of plants from Peru and China within a temperature range 8-35 °C evidenced a significant increase (p<0.05) in the degree of unsaturation during the cold season (18.2 °C on average), at the expense of oleic acid (C18:1, ω-9) and ALA (C18:3, ω3), without variation for the LA (C18:2, ω-6). Seventeen genes were found responsible for the production and accumulation of unsaturated fatty acids in the seeds during different development stages of the plants, from the pollinized flower to the mature seed (a process naturally requiring 112 days). Such content was duplicated, going from 41.3% during week four of development to 92.6% during the ripe stage, where three genes are apparently responsible of this increase, namely *SACPD* (stearoyl-acyl-carrier protein desaturase), *FAD2-2* (oleate desaturase) and *FAD3* (linoleate desaturase) (*37*).

Vašek *et al.* (*3*) conducted a research in 2017 looking to characterize the genetic diversity and population structure in 169 samples of *P. volubilis* L. from the Peruvian Amazon, performing 11 combinations of primers and the amplified fragment-length polymorphism (AFLP) method. Although it was not possible to confirm a direct relationship between the plant genetic diversity and the geographic location, this work made it possible to demonstrate the presence of nine genetic clusters corresponding to the same number of geographic sites analyzed in the San Martín region; thereby, considering the existence of distinct isolated subpopulations of this plant under the hypothesis of a possible anthropogenic influence modulating such selection (*3*).

The integrity of the plant components is a significant aspect promoting the affordability of sacha inchi for human use and consumption. Among the preservation strategies to protect the crop from microbiological contamination and pest attack on seeds and fruits, there is gamma irradiation (an alternative to chemical or thermal treatment of the plant), with high capacity for penetration and low impact on plant composition without emitting residual radiation (*12*, *38*). Gutiérrez *et al*. (*12*) recently analyzed the properties of oil extracted from sacha inchi seeds exposed to gamma radiation, such as content of fatty acids, tocopherol, peroxide value, acidity and time of oxidation induction, among others, and concluded that there was minimum effect of the treatment, reflected in decreased content of γ- and δ-tocopherols by 6.4 and 5.2-6.4%, respectively, as well as time of oxidation induction (although within that stipulated by the Peruvian norm). These results confirm that with moderate use of radiation (between 1 and 5 kGy) the physicochemical features of the plant would be maintained (*12*).

Interestingly, the oily composition of the seeds is preserved after industrial fractionation processes, favoring its availability for human consumption (*18*, *31*). Implementation of new processes like time-domain nuclear magnetic resonance (TD-NMR) offers an optimization of the oily component extraction as an alternative to the standard process of continuous extraction with organic solvents (Soxhlet method), without affecting the plant structure and allowing repeated measurements (*39*). Nuclear magnetic resonance analysis highly correlates with other approaches, like gas chromatography with flame ionization detector, which allows an accurate analysis of the content of ω-3 essential fatty acids, even in the oil enriched with ω-6 (R^2^=0.995 to 0.999) (*40*).

Likewise, the oxidative stability and concentration of essential fatty acids like ω-3 from the oily plant industrially encapsulated in ovalbumin and polysaccharide biopolymers is maintained from 1 to 1.79 years at room temperature (25 °C), a value that can be doubled with a reduction of only 5 °C (3.29 years at 20 °C) and even up to 17 days at high temperatures like 50 °C (*41*, *42*).

## PHYSIOLOGICAL EFFECTS OF THE SACHA INCHI PLANT

Sacha inchi (*Plukenetia volubilis*) is a plant with a great agroindustrial potential since it contains ω-3, -6 and -9 fatty acids, thus conferring a high nutritional value. This plant is a bioresource that can be positioned in various market segments such as dietary supplements, functional foods, cosmetics and personal care, as well in medicine, not just for the content of fatty acids but its other bioactive components summarized in the following chapters.

### Cardioprotective and immunomodulatory activities

Since the early 20th century we have known of the dietary essential fatty acids, like linoleic (ω-6) and α-linolenic (ω-3), which humans and animals cannot synthesize due to the lack of desaturases (*43*). Experiments developed by Burr and Wesson in rats (*44*) revealed the relevance that lies in the physiological functions of fatty acids, like generation of energy, plasma membrane fluidity, signal transduction, and generation of bioactive metabolites, like prostaglandins, thromboxanes, leukotrienes, lipoxins, resolvins, maresins and neuroprotectins, among others (*45*-*47*).

As mentioned before, it has been determined that PUFA content like LA and ALA in sacha inchi seeds is above 80%. These biomolecules are involved in the conformation and fluidity of the plasma membrane as well as in immune functions through the genesis of prostaglandins to mediate inflammatory response, also in cholesterolemia, brain function, and blood pressure, according to ALA-deficient animal models (*16*). Additionally, the aging process leads to reduction in the activity of desaturase enzymes that along with elongase are responsible for the synthesis of long-chain fatty acids (*43*), affecting lipid composition and function of the neuronal membrane, whose content of arachidonic acid and docosahexaenoic acid (known as DHA) depends on the hepatic synthesis (*48*).

The decrease of the LA/ALA ratio in the sacha inchi oil (lower than one) is significant, and the reduced risk of suffering biological events such as cardiovascular disease, neoplasia, severe depression, chronic inflammatory and autoimmune diseases, given the relationship of these pathologies with the presence of proinflammatory cytokines like IL-1 and leukotrienes like LTB4; the last derived from the consumption of ω-6 fatty acids. The aforementioned stems from the establishment of an optimal range for the rate *n*-6/*n*-3 fatty acids from 1:1 to 4:1, recommended for their general equilibrium in the human diet (*49*, *50*).

Gonzales *et al.* (*51*) evaluated the availability of ω-3 fatty acids in 18 healthy human individuals from 20 to 55 years of age (9 male and 9 females) after consuming 10 or 15 mL of sacha inchi oil, revealing a maximum ALA peak 4 h after intake, which was not detected in individuals consuming another vegetable oil. The maximum plasma concentration post-intake of this fatty acid was significant, (2.84±0.36) mg/mL in women and (0.94±0.57) mg/mL in men, also observing increased DHA, which reached concentrations of (2.60±0.84) and (1.00±0.38) mg/mL in women and men respectively, findings that are contrary to those obtained from individuals who consumed sunflower oil. Another study conducted for four months on 15 males and 15 females without the history of hyperlipidaemia or any other disease likely to affect lipid metabolism showed that the permanent consumption of sacha inchi oil, despite causing incipient nausea that decreased during the course of the study, is safe in terms of side effects at the renal or hepatic level, in addition to its effectiveness, given the 10% increase in plasma HDL level after its consumption during four consecutive months (*52*).

Regarding the chronic inflammatory diseases, it has been reported that ω-3 fatty acids exhibit potent immunomodulation activity attributed to the amount and type of eicosanoids derived by its consumption. Sacha inchi should also have this potential since its composition is based primarily on the content of the PUFAs (*23*-*25*). For instance, eicosapentaenoic acid (EPA) (ω-3) competes enzymatically with arachidonic acid (ω-6) for the cyclooxygenase and lipoxygenase pathways in the synthesis of prostanoids and leukotrienes, leading to diminished prostaglandin E2 (PGE_2_), thromboxane A2 (TXA_2_) and leukotriene B4 (LTB_4_) levels, involved in the processes of platelet aggregation, vasoconstriction, induction of inflammation, chemotaxis and leukocyte adhesion.

It has been reported that an optimal equilibrium between ω-6 and ω-3 fatty acids in a diet should be ranked at or near 1, as the one naturally found in *P. volubilis* L. A disbalance of this ratio has been observed in western countries, reaching 15:1 to 16.7:1 (*53*), even in the European Union, where the analysis of essential PUFA (LA and ALA) intake reveals that 52 and 77% of these countries adequately consume LA and ALA, respectively; however, in some population groups, like lactating women, adolescents, and the elderly, the intake of these essential molecules is considered inadequate (*47*). Such is the effect of this balance among essential fatty acids that a fourfold reduction of this ratio (4:1 ω-6/ω-3) reduces the mortality associated with cardiovascular diseases by up to 70%, as well as regulates the proliferation of tumors in patients with colorectal carcinoma when the ratio falls almost eight times (2.5:1), in addition to a lower risk of developing other types of neoplasms, such as breast cancer (*54*, *55*), as will be discussed ahead.

Likewise, a moderate ratio below 5:1 between ω-6 and ω-3 fatty acids controls the inflammation in patients with rheumatoid arthritis and asthma, conferring a determinant role to the balance of PUFA in the human diet in terms of maintenance (or preservation) of the state of health (*53*), all of which highlights the relevance of the contribution of ω-3 fatty acids from sources like sacha inchi in the human diet.

Although the protein content of sacha inchi seeds is approximately half that of lipids, it also exhibited anti-inflammatory properties, inhibiting the denaturation of albumin by 78.13% at 70 °C, with the advantage of its *in vitro* stability after treatment with pepsin and pancreatin (simulating gastric and duodenal digestion, respectively), which helps maintaining its biological properties after consumption (*56*).

### Antioxidant activity

The composition and the antioxidant potential of polyphenols in the seeds and also in the residual cake have been evaluated after extracting the oil by cold pressing the samples of sacha inchi (*24*). A higher content of total polyphenols and tannins was found in the hydrophilic phase of the cake, consistent with its ability to protect against oxidative damage, as well as a greater inhibition of hydrogen peroxide and a marked reduction in ferric to ferrous ion activity compared to catenin as a control, which was 14 and 29 times higher than the hydrophilic and lipophilic phases of oil, respectively (*26*).

The effect of dietary consumption of ω-3 essential fatty acids has been carefully evaluated in a murine model in terms of generation of new long-chain PUFA (LCPUFA), the activity and expression of liver desaturase and the control of oxidative stress. Rincón-Cervera *et al*. (*27*) studied male Wistar rats consuming the oil from five plants with different ALA content as the only source of lipids in the diet. Animals fed with sacha inchi oil showed a notable reduction of the ω-6/ω-3 LCPUFA from (9.24±0.6) (control group) to (0.29±0.03) g per 100 g fatty acid methyl esters (FAME) as well as a lower expression and activity of ∆-5 and ∆-6 desaturases, thus requiring biosynthesis of endogenous PUFA to a lesser extent (given the exogenous source) (*27*).

Dietary ALA intake shows a directly proportional relationship with an increased peroxisome proliferator-activated receptor alpha (PPAR-α) activation, leading to possible immunomodulation since this molecule forms a non-functional complex with the p65 subunit of nuclear factor κB (NF-κB), blocking its proinflammatory effect (*57*).

In addition, there is evidence of antioxidant potential linked to the increased level of glutathione (GSH) in plasma and diminished reduced/oxidized (GSH/GSSH) ratio. GSH is considered one of the most important antioxidant molecules, like glutathione peroxidase coenzyme (*58*), an enzyme that in that very study increased its hepatic activity, like superoxide dismutase, glutathione reductase and catalase, leading to the conclusion that ALA consumption, as shown after the diet with sacha inchi, constitutes a protection mechanism against hepatic oxidative stress (*27*).

Although the information available on the antioxidant potential of sacha inchi biocompounds is principally from seeds, it is worth highlighting that the analysis of diverse extracts obtained from leaves also showed such antioxidant potential. Various extracts like aqueous, methanolic, ethanolic, in chloroform and in hexane showed the capacity to reduce the Mo^6+^ ion from ammonium molybdate to Mo^5+^ in acid medium and in the presence of 250 µg/mL of the aforementioned extracts (generating green phosphate complex/Mo). Total antioxidant capacity, expressed in ascorbic acid equivalents (AAE), was 83.42, 89.21 and 97.76 g in hexane, chloroform and methanol extracts, respectively (*29*).

It is considered that the antioxidant potential of a plant depends on the content of its antioxidant compounds. Sacha inchi flavonoids have shown their capacity to prevent the formation and elimination of free radicals (*59*, *60*). These compounds have been identified in the hydroalcoholic extract of sacha inchi leaf (Tarapoto, Peru) and, along with tannins, have shown a significant *in vitro* inhibition of the lipid peroxidation (measured as the formation of malonaldehyde) induced by iron(II) ascorbate in hepatic tissue of rats (*Rattus rattus albinus* variety) after concomitant treatment of hepatocyte homogenate with a dosage of 70 and 140 mg/L of the extract (p<0.001), without statistical difference between dosages (*61*). Likewise, the nutshell has a high content of α-tocopherol, a molecule considered as the main active metabolite of vitamin E (*62*) and responsible for its antioxidant activity in biological systems, protecting the unsaturated fatty acids from oxidation (*63*).

Overall, the total tocopherol content in *P. volubilis* L. described by Pereira de Sousa *et al.* (*64*) in shell and seeds (average of 3.06 and 8.99 mg/100 g, respectively) is higher than that of other oleaginous plants like rye (0.1 mg/100 g), including α-, β- and γ-tocopherol (*65*), and similar to that of diverse legumes (10 mg/100 g), although in these no β-tocopherol has been detected (*66*). It should be pointed out that the values referred by Pereira de Sousa *et al.* (*64*) are even lower than the total value of vitamin E determined in other studies of oil from sacha inchi seed, as already stated in previous publications, where all the evaluated plants were from Peruvian Amazon jungle (*17*, *18*, *33*).

### Antiproliferative and antitumor activity

To date, little information is known on how the sacha inchi or its derivatives are related to neoplasia or tumor prevention. Recently, a group of researchers from Peru performed experiments based on *in vivo* colon cancer model (Wistar and Sprague-Dawely rats) with 1,2-dimethylhydrazine (*67*), a recognized inducer of colorectal carcinoma of broad experimental use with diverse rat strains (*28*, *68*-*70*). That study found a 12.5% increase in the number of individuals protected against tumor induction (without injury) in the group of rats exposed to the carcinogen and consuming sacha inchi seed oil (at a dosage of 150 µL/(kg·day)), compared with the control group, although there were no significant differences that would reveal the association between the consumption of sacha inchi oil and prevention of the formation of neoplastic lesions (*28*).

The analysis of the effect of consumption of a sacha inchi fatty acid supplement (constituted of a mixture of fatty acids, principally α-linoleic acid, ω-3 at 54.5%), in the context of antineoplastic control has yielded interesting results, as shown by the reduction of up to 2.3- and 3-fold of the tumor mass and the proliferation of Walker 256 cells in a murine model of breast cancer, respectively, consistent with the data obtained after the diet with fish oil, along with the decrease of TNF-α, IL-6 and triacylglycerides by 65, 62.5 and 50% in plasma, respectively (*30*). These findings call on the need to explore the antitumor biological activity of ALA in humans, as important component of the sacha inchi seeds. Additionally, antitumor potential of sacha inchi leaf extract has been evidenced in the decreased viability of A549 cells (human pulmonary carcinoma) and HeLa cells (human cervical carcinoma), along with a significant reduction of 48.5 and 54.3% of their proliferation after 48 h of treatment with 250 µg/mL methanol and hexane fractions, respectively. These fractions also induced early apoptosis of the HeLa cells by 10.2 and 13.3%, an effect that was higher for the aqueous fraction (17.2%) (*29*).

This activity of sacha inchi leaf extracts is interesting as they contain 5.34-10.85% polyphenols (especially the chloroform leaf extract) (*29*), compounds present in plants from the Euphorbiaceae family, like *Euphorbia* (*71*), with *in vitro* antiproliferative activity in digestive tract, although well-known for their antioxidant activity (as reducing agents) and even as metal chelators. It is considered that this same property, related to the inhibition of lipid peroxidation, protects against the onset of cancer, taking into account that within the two most important classes of polyphenols, flavonoids are the main ones found in *P. volubilis* L. (*72*). Table 1 shows the compendium of the main biological activities found in sacha inchi parts (*14*, *29*, *30*, *36*, *73*-*76*).

**Table 1:** Biological activities of sacha inchi

Biological activity	Plant part	Country	Assay outcome	Reference
Antioxidant	Seed	Peru	Seeds of 16 cultivars were assessed searching for different phytochemicals; a high variability was found in the content of the evaluated compounds. The hydrophilic and lipophilic antioxidant capacities were correlated with total phenolic and total carotenoid contents, respectively. This study positions the seed as source of polyunsaturated fatty acids, tocopherols, phytosterols and phenolic compounds with antioxidant capacity.	(*14*)
	Seed (oil)	Peru	The antioxidant activity of the lipophilic and hydrophilic extracts of the oil was measured *in vitro* by ABTS and DPPH assays. Lipophilic extract showed greater antioxidant activity using the DPPH assay than hydrophilic extract, which showed greater activity using the ABTS method.	(*36*)
	Seed (raw and honey‐coated)	Peru	Several approaches (open boiling, pressure boiling, low and high temperature roasting and honey roasting) were applied to kernels to assess the variations in the total phenolic content. The result of the DPPH assay was influenced by process temperature and water activity of the seeds.	(*73*)
	Leaf (leaf extract and leaf extract-based silver nanoparticles)	Ecuador	The antioxidant effect of AgNPs (silver nanoparticles) was higher than of leaf extracts against the DPPH radicals. Maximum radical scavenging activity was 22.5% in 0.6 mL of AgNPs whereas 19% in 1.0 mL of leaf extracts.	(*74*)
Antidyslipidemic	Seed (roasted)	Peru	The effect of the intake of 30 g sacha inchi seeds per day for 6 weeks was assessed on 28 volunteers. The control group received 30 g confit wheat (*Triticum aestivum*). A reduction in cholesterol, triglycerides and LDL levels was observed, as well as an increase in HDL levels.	(*75*)
	Seed (oil)	Peru	This experimental work sought to know the effect, effective dose and side effects of sacha inchi oil in the lipid profile of 24 patients with hypercholesterolaemia. The participants were randomized to receive 5 or 10 mL of an oil suspension for four months. Intake of the oil resulted in a drop in mean total cholesterol and non-esterified fatty acid values with c-HDL elevation in both groups.	(*76*)
Antitumoral	Leaf (leaf extracts)	Brazil	HeLa (cervix) and A549 (lung) tumor cell lines were treated with several leaf extracts. The methanol and hexane compounds were able to reduce the proliferation of HeLa cells up to 54.3 and 48.5%, respectively.	(*29*)
	Seed (oil)	Peru	Seed oil was shown to have potential anticancer activity in Walker 256 tumor-bearing rats. A sacha inchi oil-based diet (1 g/kg body mass, daily, for 4 weeks) reduced tumor mass and proliferation of Walker 256 tumor cells *ex vivo*. This assay also identified an increased lipoperoxidation in Walker 256 tumor tissues as well a reduction of the glycaemia, triglycerides and inflammatory cytokine plasma levels.	(*30*)

This table summarizes experimental trials focused on evidencing three clinical or biological applications related to certain parts of the sacha inchi plant. In relation to the origin of the crop, it is evident that most of the studies come from South America, specifically Peru. ABTS=2,2’-azinobis-(3-ethylbenzothiazoline-6-sulfonate) assay, DPPH=2,2-diphenyl-1-picryl-hydrazyl-hydrate assay

## TECHNOLOGY AND SACHA INCHI

Currently, some technological tools are known that facilitate the use of the useful components of sacha inchi, such as nanoparticles and microencapsulation. One of the new therapeutic trends in the oncology, for example, is related to the use of tiny particles with special features in terms of specificity and biological safety. The peculiarity of these molecules (nanoparticles) is mainly based on their small size and the versatility to act as transporters of any type of element and to interact with biomolecules present in different cellular locations, with properties such as a size smaller than 100 nm, as well as a contrast between rigidity and flexibility, giving them use in medicine, among other areas (*77*, *78*).

The nanoparticles have been proven as an alternative in the distribution of drugs in the treatment of cancer, and among the most evaluated ones are gold, silver and iron nanoparticles due to their outstanding properties (*79*). It has been observed that these molecules exhibit stability and easy entry into the cell, in addition to an acceptable biocompatibility. However, the main scientific attraction is that they are biologically inert and non-toxic and can also be synthesized through two mechanisms: chemically, a process that involves the use of toxic chemical agents, prolonged synthesis protocols and physical processes that affect the stability of the nanoparticles; and through the use of plants (*80*, *81*), a process known as green chemistry, which is cost-effective, environmentally friendly and scalable, especially for low-income countries.

There are several approaches focused on demonstrating the effectiveness of nanoparticles synthesized from plant extracts, revealing both their microbicidal and tumoricidal capacities. Ezhilarasi *et al*. (*82*) used nickel oxide nanoparticles prepared from extracts of *Moringa oleifera*, demonstrating the inhibitory effect of *in vitro* proliferation on HT-29 colon tumor cells, postulating in this way the potential of this green technology in biomedical treatments. An interesting finding regarding biosecurity was observed in the performance of gold nanoparticles conjugated with extracts also obtained from this plant, one of the most studied in this regard. This assay demonstrated the absence of cytotoxicity of the nanoparticles towards blood mononuclear cells, but in turn showed proliferative inhibitory effects in A459 and SNO tumor lines through the induction of apoptotic mechanisms (*83*), confirming the selectivity of the phytonanoparticles. As for the synthesis of phytonanoparticles using sacha inchi, the conjugation of these extracts with silver particles has been achieved, which have shown antioxidant effects (Table 1) (*74*).

An even more ecological and cost-effective method of synthesis was developed using sunlight as the source of energy, which catalyzes the formation of gold particles together with the sacha inchi oil extract (*84*). So far, there is no evidence of the application of nanoparticles based on sacha inchi in studies of basic experimentation regarding their behavior as an element with antitumor properties, opening a potential exploratory market for this purpose.

On the other hand, the food industry favors microencapsulation technology through the enrichment of foods with PUFAs such as ω-3 and -6 from sacha inchi, while preserving the characteristics of these essential fatty acids, improving their oxidative stability, as mentioned previously (*41*, *42*), and also by generating emulsions and combining spray drying and spray chilling methods, with wall materials such as skimmed milk powder, acacia gum, mixture of grape juice and acacia gum, and hydrogenated palm oil, with acceptable organoleptic properties (*85*).

The microencapsulation method by spray drying continues to show high efficiency and oxidative protection of *Plukenetia volubilis* L. oil, among other plant species, in the field of functional foods, especially when using modified starch (Hi-Cap) as wall material, with an encapsulation efficiency of 93.3% compared to the range of 61.1-73.0% obtained with other wall materials such as maltodextrin, gum Arabic, whey protein concentrate or a mixture of these; likewise, a lower humidity and a notably higher half-life of 144.3 days at 25 °C compared to an average of 79.9-84.1 days for the other materials (*86*).

In the plant seed oil microencapsulation technology, other wall materials such as ovalbumin (protein) and sodium alginate (polysaccharide) have been used jointly as biopolymers by complex coacervation process, providing resistance to high temperatures (approx. 190 °C) and gastric digestion (given its pH=3.8 and the polysaccharide wall), also with an efficiency close to 95% in the encapsulation process and a higher bioavailability of the oil for its intestinal absorption (*87*).

Very recently, this last microencapsulation process has been optimized by adding a third component (tannic acid) to the protein-polysaccharide wall (ovalbumin-pectin), with a contrasting effect since the nutritional value of the capsules seems to be increased, although the encapsulation efficiency did not exceed 80% (*88*). The use of this acid not only replaces the use of other compounds considered toxic such as glutaraldehyde and formaldehyde to facilitate the interaction between ovalbumin biopolymers and polysaccharides (*89*), but because it is constituted by high molecular mass polyphenols, it provides an additional contribution as a result of its antioxidant value (*90*, *91*).

## POTENTIAL FOR RESEARCH

Besides the biological activity described for sacha inchi components, the antimicrobial effect, emollient activity and absorption of heavy metals have been reported recently (*25*, *29*, *92*). In the first case, the effect of the plant seed oil has been explored on the adherence of skin commensal bacteria, *Staphylococcus aureus*, starting from the traditional recognized use (empirical) of the plant to treat cutaneous wounds, besides humectation and scarring (*93*, *94*).

The bactericidal potential of Amazonian sacha inchi extra virgin oil has been assessed (with standard content of 84% PUFAs, 48% ALA and 35% LA) through inhibition of bacterial growth after treatment of 10^8^ CFU/mL with the oil during 48 h and at corporal temperature conditions, with respect to the control group in a medium without the oil and phenol as positive control; however, such effect was not observed, since a survival of approx. 90% of the microorganisms was evidenced in the experiment. In contrast, protective or preventive ((39.2±3.4) %) and curative ((33.9±1.8) %) outcomes were found against adherence of *S. aureus* based on the oil treatment of human keratinocytes and skin explants in the presence of the microorganism; an effect possibly attributed to the content of ω-3 and ω-6 fatty acids in this oil, discarding its possible direct damage (cytotoxicity) to the epithelial cells (*93*).

Due to the scarce information available, it is important to explore in depth the possible antimicrobial potential *of P. volubilis* L., since it is not yet catalogued within the group of 221 medicinal plants with known antifungal, antiparasitic, antiviral or bacteriostatic activity. Besides, it is extremely important to find biological alternatives, in this case of plant origin, to counteract the current increase in bacterial resistance, which represents a need, as well as a clear opportunity for social welfare.

The bioremediation effect of sacha inchi shells has been exemplified in the bioabsorption of Pb^2+^ and Cu^2+^ ions. Treatment of aqueous solutions containing heavy metals with biomass based on the seed shell triturate under conditions of acidic pH and 323 K° revealed the increase of 15.72% of Pb^2+^ and 6.33% of Cu^2+^ (by mass) on the surface of the shell biomass after the biosorption treatment, as the evidence of the bioremediation effect (*92*). This biological activity recently attributed to *P. volubilis* L. components represents a potential use and benefit, given that the process used to obtain them is simple and cost-effective with the vast availability of the plant waste products.

The properties of sacha inchi as an emollient containing saponin have been assessed in a study that recruited infants from 4 to 8 years of age with atopic dermatitis. This study sought to favor the reestablishment of the affected skin given the known imbalance of fatty acids, a key feature in this pathology, finding a significant improvement of the inflammation and pruritus observed in the group treated during two months with the plant oil (p=0.0004), with respect to the control group (placebo), an effect that remained stable over time one month after suspending the treatment (p=0.002) (*95*). These observations give an encouraging opportunity to treat this chronic condition without a known cure, as well as a panorama of constant inquiry of the still unknown benefits of the Amazonian plant.

Given the benefit to human health attributed to the consumption of ω-3 fatty acids, Peru is currently planning to expand the use of food sources that contain them, favoring their dietary intake through strategies like feeding guinea pigs, poultry and chickens with a combination of sacha inchi oil and fish, which enrich their meat with these lipids (*16*). Such types of alternatives could impact positively the maintenance of human health since fish oil is recognized as a source of ω-3 fatty acids, like EPA and DHA, due to their higher degree of unsaturation than of ALA. These PUFAs contribute, through their incorporation, to a variety of processes such as regulation of the inflammatory immune response, blood pressure (which has been lower in individuals who consume it with respect to the control group without consumption) (*96*), reduction of triacylglyceride levels in the circulation, prevention of neurodegenerative and neuropsychiatric disorder, maintenance of memory and visual function (*97*-*99*).

However, the high content of essential fatty acids (EFA) from the sacha inchi seed oil, especially at the expense of ALA, will permit the production of other types of LCPUFA, like EPA and DHA, in the organism (its metabolites) with all its attributed benefits (*43*, *100*, *101*).

Sources of high daily impact are represented by milk and dairy products, like yoghurt, already supplemented with components rich in linoleic fatty acid as for example fish and sunflower oil, canola and soy, showing a moderate increase of unsaturated fatty acid content (*102*-*104*). Supplementation of yoghurt with sacha inchi seeds has been studied recently, revealing an increase of the levels of ALA and LA of 25- and 50-fold respectively, from an average PUFA content of 3.60 to 81.51% in the modified product compared to the control, concomitant with reduced content of saturated fatty acids (palmitic and stearic) from 76 to 84% and sensory acceptance by >70% consumers (volunteers) (*105*).

## CONCLUSIONS AND RECOMMENDATIONS

*Plukenetia volubilis* L. (sacha inchi) is part of a selected list of the most promising plants in Peruvian traditional medicine, attributed to the location where it has been described historically, jointly with *Smallanthus sonchifolius* (yacon root), *Croton lechleri* (dragon’s blood), *Uncaria tomentosa/U. guianensis* (cat’s claw), *Lepidium meyenii* (maca root), *Physalis peruviana* (cape gooseberry), *Minthostachys mollis* (muña), *Notholaena nivea* (cuti-cuti), *Maytenus macrocarpa* (chuchuhuasi), *Dracontium loretense* (jergon sacha), *Gentianella nitida* (hercampuri) and *Zea mays* (purple corn) (*35*), highlighting, among other features, the high nutritional value and antioxidant potential of the phenolic components of its seeds (mostly tannin type, 93.1%) (*25*). The presence of polyphenols from this plant has been also reported in its leaves (*26*, *29*).

It is considered that fatty acids with higher fraction of PUFA in sacha inchi are beneficial to human health due to their antiatherogenic, antithrombogenic and hypocholesterolemic effects (*64*), besides having a high nutritional value, projecting it as a promising crop with a potential for cost-effective production that will allow considering alternatives to substitute illegal crops in the Putumayo region in Colombia, for example by increasing the social impact of this plant (*23*, *29*).

Regarding the usefulness of its components, there is evidence of antitumor activity of sacha inchi seed oil and aqueous and organic leaf extracts, to which we add the finding of the stimulant effect of the latter on the proliferation of normal cells, confirmed by the increase of approx. 175% of the 3T3 cells (mouse fibroblasts) (*29*). This opens the door for a deep search of other potential uses of this plant, for example, in tissue regeneration, which requires the use of human cells.

The genetic and population diversity of *P. volubilis* L. has been described dependent on the geographic region of origin, implying the need to study the biological properties of the plant, bearing in mind the region from which it is obtained, as a constant research activity in favor of recognizing its potential and new evidence of usefulness to benefit human health.
